# Identification of New Biological Pathways Involved in Skin Aging From the Analysis of French Women Genome-Wide Data

**DOI:** 10.3389/fgene.2022.836581

**Published:** 2022-03-24

**Authors:** Myriam Rahmouni, Vincent Laville, Jean-Louis Spadoni, Randa Jdid, Leopold Eckhart, Florian Gruber, Taoufik Labib, Cedric Coulonges, Wassila Carpentier, Julie Latreille, Frederique Morizot, Erwin Tschachler, Khaled Ezzedine, Sigrid Le Clerc, Jean-François Zagury

**Affiliations:** ^1^ Équipe Génomique, Bioinformatique et Chimie Moléculaire (EA 7528), Conservatoire National des Arts et Métiers, HESAM Université, Paris, France; ^2^ Chanel R&T, Department of Skin Knowledge and Women Beauty, Pantin, France; ^3^ Department of Dermatology, Medical University of Vienna, Vienna, Austria; ^4^ Christian Doppler Laboratory for Skin Multimodal Analytical Imaging of Aging and Senescence (SKINMAGINE), Medical University of Vienna, Vienna, Austria; ^5^ Plate-Forme Post-Génomique P3S, Hôpital Pitié-Salpêtrière, Paris, France; ^6^ Department of Dermatology, Hôpital Henri Mondor and EA 7379 EPIDERM, Créteil, France

**Keywords:** SNP, skin, aging, pathway, GWAS

## Abstract

Skin aging is an ineluctable process leading to the progressive loss of tissue integrity and is characterized by various outcomes such as wrinkling and sagging. Researchers have identified impacting environmental factors (sun exposure, smoking, etc.) and several molecular mechanisms leading to skin aging. We have previously performed genome-wide association studies (GWAS) in 502 very-well characterized French women, looking for associations with four major outcomes of skin aging, namely, photoaging, solar lentigines, wrinkling, and sagging, and this has led to new insights into the molecular mechanisms of skin aging. Since individual SNP associations in GWAS explain only a small fraction of the genetic impact in complex polygenic phenotypes, we have made the integration of these genotypes into the reference Kegg biological pathways and looked for associations by the gene set enrichment analysis (GSEA) approach. 106 pathways were tested for association with the four outcomes of skin aging. This biological pathway analysis revealed new relevant pathways and genes, some likely specific of skin aging such as the *WNT7B* and *PRKCA* genes in the “melanogenesis” pathway and some likely involved in global aging such as the *DDB1* gene in the “nucleotide excision repair” pathway, not picked up in the previously published GWAS. Overall, our results suggest that the four outcomes of skin aging possess specific molecular mechanisms such as the “proteasome” and “mTOR signaling pathway” but may also share common molecular mechanisms such as “nucleotide excision repair.”

## Introduction

Aging is an ineluctable process leading to the progressive loss of tissue integrity. Skin aging is the most obvious feature of this process and can be characterized by several outcomes such as wrinkling, sagging, and pigmented spots (Raschke and Elsner, 2010). A great effort has been made to determine environmental factors and molecular mechanisms involved in skin aging. The environment greatly impacts skin aging (Guyuron et al., 2009), especially UV exposure that induces a particular phenotype of skin aging called photoaging (Rabe et al., 2006; Yaar and Gilchrest, 2007). However, in this environment-dependent context, genetic factors may also exert a modulatory role. Indeed, heritability studies performed on Caucasian twin subjects suggest a genetic component to the variation of skin aging features that support the use of genome-wide analysis studies (GWAS) on skin aging: 55% for facial wrinkling, 41% for pigmented spots, and 61% for sagging eyelids (Gunn et al., 2009; Jacobs et al., 2014). Over the last 3 years, several GWAS focusing on skin aging have been published and several genes associated with skin aging have been identified, some of which have revealed new molecular mechanisms involved in skin aging: a first GWAS identified an association between the *STXBP5L* gene and facial photoaging (Le Clerc et al., 2013), a GWAS focusing on sagging eyelids highlighted the possible role of the *TGIF1* gene (Jacobs et al., 2014), two GWAS performed on skin youthfulness unraveled, respectively, the *KCND2*, *DIAPH1,* and *EDEM1* genes (Chang et al., 2014) and the *MC1R* genes (Liu et al., 2016), and a GWAS identified associations between the four skin color genes *MC1R*, *IRF4*, *BNC2*, and *RALY*/*ASIP* and facial pigmented spots (Jacobs et al., 2015; Jacobs et al., 2015), whereas a second GWAS focusing on facial solar lentigines emphasized the possible role of the *HLA* region in their occurrence (Laville et al., 2016).

However, it has now been well established that individual SNP associations in GWAS do not represent the whole impact of genetics in complex polygenic phenotypes such as skin aging (Maher, 2008) because of the lack of power and multiple testing. These individual SNP associations only pick up specific loci with very strong influence on the phenotype, and they often stop short of providing solid biological mechanisms. To overcome these limitations, strategies that integrate biological knowledge have been developed to look for new associations buried under the significance threshold (Wang et al., 2010a). One of the most popular approaches is to look at the level of biological pathways associated with a phenotype, using the gene set enrichment analysis (GSEA) (Wang et al., 2007). Biological pathways are defined as a set of interacting molecules and reactions between entities (nucleic acid, protein, complex, etc.) forming a biological network and have been classified in categories. For example, “melanogenesis” and “glycolysis” are biological pathways belonging to the “metabolism” category. In the GSEA approach, a pathway is represented by a set of genes involved in a particular biological network. The underlying concept is that various genes involved in the same biological pathway may contain genetic variants, each having a moderate effect that will not be picked up using the GWAS significance threshold. Identifying associations between a complex trait and biological pathways will, thus, be of great interest because it will provide more functional insights to understand the underlying molecular mechanisms and, as a consequence, likely provide further therapeutic or diagnostic targets for specific aspects of skin aging.

In this work, we used a set of 502 well-characterized Caucasian women from the SU.VI.MAX cohort that were genotyped with the Illumina HumanOmni1-Quad BeadChips leading to 795,063 SNPs after quality control. We looked for new associations at the level of biological pathways for four major outcomes of skin aging, namely, photoaging, solar lentigines, wrinkling, and sagging. For each outcome, we performed a classical GWAS using a regression adjusted for various confounding factors, and we then treated the data in the GSEA pipeline to look for pathway associations.

## Materials and Methods

### Study Design and Population

This population was previously described in detail (Le Clerc et al., 2013). Briefly, in the autumn/winter of 2002–2003, 570 out of the 2,257 middle-aged women living in the Paris area from the SU.VI.MAX cohort (Hercberg et al., 1998) agreed to participate in a research study on skin aging and provided informed consent. The protocol was approved by the Hospital Medicals Ethics Committee of Paris-Cochin (CCPPRB no. 706) and the “*Commission Nationale de l’Informatique et des Libertés*” (CNIL no. 334641). The study was conducted according to the Declaration of Helsinski Principles. Each participant completed a self-administered questionnaire related to lifetime sun exposure behavior, and three standardized high-resolution digital images (2,008 × 3,032 pixels) of the face were taken under normalized lighting conditions (one frontal view of the face and one of each profile), using a Kodak DCS 760 digital camera with a 105 mm camera lens (Kodak, Paris, France). Moreover, a blood sample was collected from each woman.

### Outcome Variables: Phenotype Analyzed

After image acquisition, several skin aging features were assessed by a dermatologist. The Photoaging was evaluated using a scale developed by Larnier et al., (1994), as described in a previous study (Le Clerc et al., 2013). Wrinkles were visually graded by a dermatologist on different areas of the face using a scale from photographs and then a global wrinkling score on the face was computed using principal component analysis and linear regression. The same methodology has been applied to obtain a global score of sagging and a global score of solar lentigines on the face. The photoaging scale and the three outcome scores were analyzed in this study.

### Covariates Used for the Statistical Analysis

Several characteristics susceptible to play a role in the onset of the different outcomes analyzed were taken into account: age (in years), body mass index (BMI; in kg.m^−2^), smoking habits (never, former, and current), and hormonal status (nonmenopausal, menopausal with hormone replacement therapy, and menopausal without hormone replacement therapy). BMI was categorized as underweight or normal (BMI < 25 kg m^−2^), overweight (25 ≤ BMI < 30 kg m^−2^), or obese (BMI ≥ 30 kg m^−2^) according to the World Health Organization (WHO) recommendations (WHO, 1995) Moreover, lifetime sun exposure intensity was estimated by a score based on data collected by a self-reported questionnaire (Ezzedine et al., 2013). The design, validation, and description of this score have been described previously (Guinot et al., 2001).

### Genotyping Method

The genotyping method has already been described in detail (Le Clerc et al., 2013). The 529 women were genotyped using Illumina Infinium HumanOmni1-Quad BeadChips (Illumina, San Diego, CA) that contain 1,140,419 markers, and a sample of 250 ng of ADN by individual was used to obtain genotypes. For the analysis, we considered only SNPs, consequently excluding the copy-number variations that represented 91,706 markers on the HumanOmni1-Quad BeadChips. Moreover, 2,182 SNPs located on the Y chromosome were removed.

### Quality Control

The quality control steps have been described in a previous study (Le Clerc et al., 2013). Briefly, nine samples with a call rate (percentage of SNPs genotyped by sample) of <95% in the Illumina clusters were removed. The SNPs with a call frequency (percentage of samples genotyped by SNP) of <99% were reclustered and, after that, samples with a call rate of <98% were deleted. In total, after these quality control steps, 56,479 SNPs with a call frequency of <98% (2% of missing data) were excluded. The Hardy–Weinberg equilibrium analysis was performed for each SNP by using an exact statistical test implemented in the PLINK software (Purcell et al., 2007). Thus, 3,866 SNPs, which were not in the Hardy–Weinberg equilibrium (*p* < 1 × 10^–3^), were rejected. Finally, we removed 191,123 SNPs with minor allele frequency <1% to avoid errors of genotyping, leaving a total of 795,063 SNPs.

### Identification of Population Stratification

To correct for possible population stratification, genotypes were analyzed using EIGENSTRAT utility of the EIGENSOFT package version 4.2 (Price et al., 2006). The two first pass with the EIGENSTRAT software pointed out 18 outliers, who were removed from further analyses. Then, a third pass without outliers was performed to determine the Eigen vectors. In the statistical analysis, we used the top two Eigen vectors as covariates to correct for population substructure in the association analyses.

### SNP-Gene and Gene-Pathway Mappings

The 795,063 SNPs were assigned to genes according to their physical position in the hg19 build using ANNOVAR (Wang et al., 2010b). To take into account promoting and regulatory regions, the genes were enlarged 10 kb upstream and downstream. One hundred eighty six KEGG pathways were downloaded from MSigDB (Subramanian et al., 2005). To reduce bias inherent to the different number of genes in the pathways, we only analyzed pathways composed of at least 20 genes and 200 genes maximum. Finally, 364,087 SNPs were mapped in 20,161 genes gathered in 160 pathways.

### Statistical Analysis

We first performed GWAS on each of the four outcomes as described in a previous work (Le Clerc et al., 2013). Briefly, for each outcome, the association between each SNP and the outcome was measured using a linear regression with a genotypic model adjusted on the first two Eigenstrat principal components and the potential confounding factors (smoking habits, BMI, hormonal status, lifetime sun exposure intensity, and age) using the PLINK software (Purcell et al., 2007). Then, we looked for associations between the biological pathways and each outcome by performing the gene set enrichment analysis (GSEA) implemented in the GenGen software suite (Wang et al., 2007; 2010a). 5,000 *p*-value permutations were performed for the normalization of the enrichment score. The significance and correction for multiple tests were based on the 5,000 permutations and finally assessed using the false discovery rate (FDR). Pathways with an FDR below 0.05 were considered as significantly associated with the outcome analyzed and pathways with an FDR between 0.05 and 0.25 were considered as of interest. Additionally, we investigated pathways having FDR <0.25 by looking at their top ranking genes, and for that, we picked up the genes of these pathways for which the associated *p* values were < 0.01.

### Enrichment Analysis

According to the classification of the KEGG pathways, the pathways are assigned to six categories: “environmental information processing,” “cellular processes,” “metabolism,” “organismal system,” “genetic information processing,” and “human disease.” For each outcome, we looked for an enrichment of significant pathways (FDR < 0.25) in the six categories compared to the distribution of these categories in the whole KEGG database. We used a Fisher’s exact test based on hypergeometric distributions to compare the distribution of the significant pathways in each category with the distribution of the pathways of the whole KEGG database in these categories.

## Results

We looked for pathway associations using the GSEA pipeline based on 160 pathways selected from KEGG database (Kanehisa et al., 2017) and the four outcomes of skin aging: photoaging, solar lentigines, wrinkling, and sagging. We first selected the pathways with an FDR < 0.05, and then, the pathways with an FDR < 0.25 in common with two or more outcomes.

For the four phenotypes analyzed, five pathways reached the 0.05 FDR threshold. The wrinkling phenotype was associated with the “nucleotide excision repair” (*P* = 2 × 10^−4^, FDR = 0.02) and “proteasome” (*P* = 8 × 10^−4^, FDR = 0.04) pathways. For these two pathways selected by GSEA, it was interesting to look at their top ranked genes (with associated *p* values < 0.01), they were, respectively, *XPC*, *MNAT1*, and *DDB1,* and *PSMB3*, *PSMA1*, *PSMC2*, *PSMD2*, *PSMB5*, and *PSMD4* (Supplementary Table S1). The sagging outcome exhibited three pathways with an FDR below 0.05: the “amino sugar and nucleotide sugar metabolism” pathway (*P* = 2 × 10^−4^, FDR = 0.04), the “mTOR signaling pathway” (*P* = 8 × 10^−4^, FDR = 0.05), and the “nucleotide excision repair” pathway (*P* = 8 × 10^−4^, FDR = 0.05). The top ranked genes of these pathways are also presented in Supplementary Table S1. Interestingly, the sagging and wrinkling phenotypes were both associated with “nucleotide excision repair,” and the only top ranked gene in common was *DDB1*. No pathway association with an FDR below 0.05 was found for the photoaging or solar lentigine phenotypes.

**FIGURE 1:**
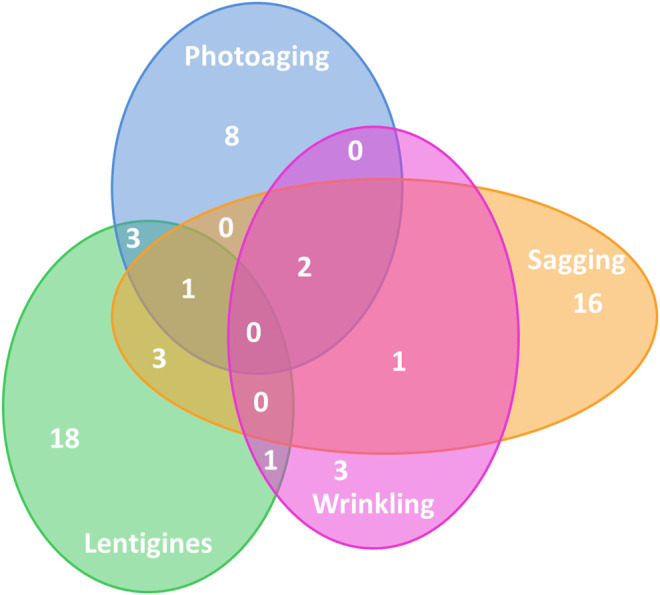
Distribution of significant pathways in KEGG database categories. The four circles represent the most significant pathways (FDR < 0.25) for the four outcomes. The number of pathways is indicated in the corresponding area with a total of 14 significant pathways for photoaging, 7 for wrinkles, 23 for sagging, and 25 for lentigines.

We then looked for pathways exhibiting an FDR < 0.25, in common with two or more outcomes (Figure 1, Supplementary Tables S2–S5). Eight pathways were shared between two phenotypes: “arrhythmogenic right ventricular cardiomyopathy (ARVC),” “long-term potentiation,” “mTOR signaling pathway,” “phosphatidylinositol signaling system,” “regulation of actin cytoskeleton,” “ribosome,” “thyroid cancer,” and “vasopressin regulated water reabsorption.” No pathway exhibited association with the four aging phenotypes, but three pathways were associated with three phenotypes: “melanogenesis” (Supplementary Figure S1) with photoaging, lentigines, and sagging, and “primary immunodeficiency” (Supplementary Figure S2) and “nucleotide excision repair” with photoaging, sagging, and wrinkles. For the top ranked genes (*p* value < 0.01) of “primary immunodeficiency,” no gene was found in common for the three outcomes. In contrast, the *DDB1* gene for “nucleotide excision repair” and the *WNT7B* and *PRKCA* genes for “melanogenesis” were associated with the three outcomes. We further investigated the top-ranked genes of all these pathways in common between phenotypes. Interestingly, for the melanogenesis pathway, the top-ranked genes encoded proteins having identical functions or interacting with each other (Supplementary Table S6; Supplementary Figure S1).

**FIGURE 2:**
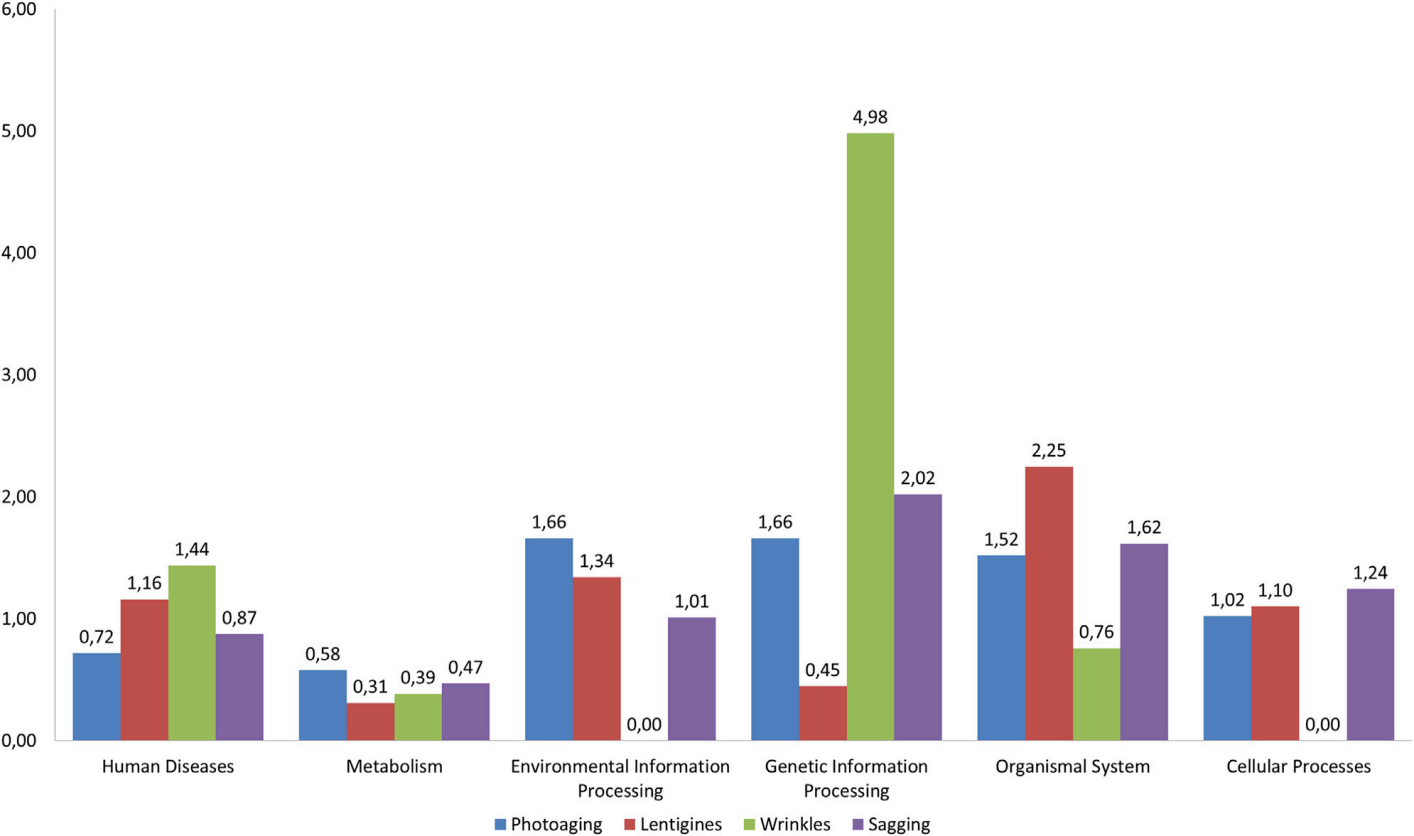
Diagram of pathways of interest for the four outcomes. For each phenotype, the most significant pathways (FDR < 25%) have been considered. There were 14 pathways for photoaging, 7 pathways for wrinkles, 23 pathways for sagging, and 26 for lentigines (see Figure 1). These pathways were assigned to one of the six KEGG categories (shown in abscissa of the figure). The ratio (R) of the percentage of significant pathways in a category for each phenotype to the percentage of pathways in a category for KEGG was computed (shown in the Y-axis).

For each outcome, we performed an enrichment analysis by comparing the distribution of significant pathways (with FDR < 0.25) in the six categories of KEGG (“environmental information processing,” “cellular processes,” “metabolism,” “organismal system,” “genetic information processing,” and, “human disease”) to the pathway distribution in the categories in the whole KEGG database. Figure 2 illustrates this analysis showing the ratio (R) of the percentage of significant pathways in a category found for each outcome to the percentage of pathways in that category over the whole KEGG database. Using Fisher’s exact test based on hypergeometric distributions, we have computed the statistical significance of the enrichments observed in Figure 2 (see Methods). For wrinkles, “genetic information processing” pathways were over-represented (R = 4.98, *p* = 1.49 × 10^–2^). “Organismal system” was over-represented for solar lentigines with nearly half (42.31%, see Supplementary Figure S3) of the associated pathways in this category (R = 2.25, *p* = 2.3 × 10^–3^, Figure 2). Moreover, 44.6% of the pathways of the category “organismal system” found for the solar lentigines phenotype belonged to the sub-category, “immune system,” whereas photoaging, sagging, and wrinkling phenotypes obtained, respectively, 0, 16.6, and 0% of associated pathways in this sub-category (data not shown). Finally, the “metabolism” category is underrepresented for the lentigine outcomes compared to the KEGG database with R = 0.31 and *p* = 2.1 × 10^–3^ (Figure 2).

## Discussion

We have performed a pathway-based genome-wide association study on four skin aging outcomes, namely, photoaging, solar lentigines, sagging, and wrinkling. The pathway-based approach looks for associations with a set of genes involved in the same biological function (*i.e*., pathway). Therefore, by taking into account prior biological knowledge, this approach leads to a more comprehensive integration of underlying biological mechanisms than the usual GWAS single SNP approach. We first investigated the results for individual outcomes and then looked for shared pathways, in order to underline specific and shared mechanisms.

The main results for the wrinkles were the “nucleotide excision repair” and “proteasome” pathways. These two pathways are directly related to the maintenance of DNA and protein integrity, and are thus highly relevant, since accumulation of genetic damage throughout life (López-Otín et al., 2013; Moskalev et al., 2013), impairment of protein homeostasis, and proteostasis (Powers et al., 2009; López-Otín et al., 2013) play a key role in aging and also in skin cancer, the ultimate step of skin aging. The investigation of the top-ranked genes of the “nucleotide excision repair” pathway yielded relevant biological evidence: *XPC* and *DDB1* are involved in Xedoderma pigmentosum (XP), which is a pigmentary skin disorder characterized by solar hypersensitivity of the skin with dermal atrophy and high predisposition for developing cutaneous melanoma and squamous cell carcinoma of the skin (Li et al., 1993; Puumalainen et al., 2016). The symptom of XP reflects a deficiency of the “nucleotide excision repair” pathway in the maintenance of the genome stability in human skin (Marteijn et al., 2014), and could be used as a model of accelerated photoaging, emphasizing a putative role of *XPC* and *DDB1*. In contrast, no gene in the “proteasome” pathway was directly linked with aging, although deeper investigation of this pathway is of interest.

Regarding the sagging phenotype, the “amino sugar and nucleotide sugar metabolism” pathway yielded the best FDR and the three most associated genes were *PMM2*, *GFPT2,* and *GMDS*. *PMM2* is involved in a metabolism disorder linked with abnormal fat distribution and skin wrinkling (Kouwenberg et al., 2014). A decrease in the expression of the *GFPT2* and *GMDS* genes is observed with aging (Craig et al., 2015). Additionally, sagging was also associated with the “nucleotide excision repair” pathway, pointing out several genes, namely *DDB1* and *RAD23B,* which are both involved in XP (Kapetanaki et al., 2006; Petruseva et al., 2009), and *ERCC8* associated with the Cokayne syndrome (Ridley et al., 2005), another disease characterized by sun hypersensitivity and progeroid appearance, but without an association with skin cancer. Finally, sagging was associated with the “mTOR signaling pathway.” Strikingly, the inhibition of the mTOR pathway has been experimentally linked to an extension of lifespan in animal models and protects against a growing list of age-related pathologies (Johnson et al., 2013).

Three pathways were commonly associated with three outcomes. In detail, the “melanogenesis” pathway was associated with photoaging, solar lentigines, and sagging, whereas the “nucleotide excision repair” and “primary immunodeficiency” pathways were associated with photoaging, sagging, and wrinkling. Among the top-ranked genes, three genes were shared by three outcomes: *DDB1* belonging to the “nucleotide excision repair” pathway, and *WNT7B* and *PRKCA* belonging to “melanogenesis.” The *DDB1* gene encodes a protein belonging to the UV–DDB (ultraviolet radiation–DNA damage-binding protein) complex, which directly binds to UV-radiation-induced lesions and functions as an auxiliary damage-recognition factor (Marteijn et al., 2014). Hence, *DDB1* defect/modulation might be involved in photoaging, sagging, and wrinkling in response to UV exposure. The *WNT7B* gene is a member of the WNT gene family, known to encode proteins that have been implicated in oncogenesis and in several developmental processes, including the regulation of cell fate and patterning during embryogenesis. The *PRKCA* gene encodes a protein kinase, belonging to the PKC family, known to phosphorylate a wide variety of protein targets and involved in diverse cellular signaling pathways. Interestingly, for the “melanogenesis” pathway common for three outcomes, the top-ranked genes of the three outcomes encoded proteins with identical functions or interacting with each other. For example, we found several genes encoding adenylate cyclases, and they contribute to the same function in the pathway (Supplementary Table S6). A functional investigation of these proteins and their role in the “melanogenesis” pathway could explain the differences between the three outcomes and lead to new insights into their mechanisms of action.

Beyond analyzing the pathways one by one, it seems important to have a global overview of these pathways and shared mechanisms as skin aging is probably a complex process involving a combination of pathways. Strikingly, the “metabolism” category was poorly represented in the four outcomes (especially when compared with its KEGG distribution). In contrast, we observed an over-representation of pathways from the “genetic information processing” category associated with wrinkles (compared with KEGG). This observation may recall the influence of UV exposure on aging for the induction of DNA damage and development of wrinkles (Moskalev et al., 2013). Finally, “organismal system” was the most represented category for lentigines (Supplementary Figure S3, Figure 2). For the solar lentigines phenotype, the subclass “immune system” represented 45.4% of the pathways from “organismal system” (five pathways), underlying a putative involvement of the immune system for the solar lentigines in accordance with previous reports (Aoki et al., 2007; Laville et al., 2016).

The pathway-based approach is powerful but has a few limitations. For this analysis, we used the pathways from the KEGG database, and since the understanding of human gene function is incomplete and some genes are not yet characterized, the curated pathways of the database may not be fully representative of all the biological networks. Additionally, in GSEA, the best SNP (weaker *p* value from the GWAS analysis) is chosen to represent a gene, and this approach is limitative knowing that several variants in the same gene may contribute to the overall association signal. However, some of these limitations have been overcome by several studies (Segrè et al., 2010; Mei et al., 2016; Marczyk et al., 2021).

In conclusion, this is the first pathway-based analysis on skin aging performed on a cohort of 502 very-well-characterized women. Our study points to specific and/or shared highly relevant pathways in photoaging, solar lentigines, wrinkling, and sagging phenotypes. Results of this work may help to further identify diagnostic or therapeutic targets for specific skin aging aspects. Interestingly, some of the identified pathways may be shared by aging features that involve other organs. As for any large-scale study, a replication in other cohorts will be needed to confirm these results.

## Data Availability

The original contributions presented in the study are included in the article/Supplementary Material, further inquiries can be directed to the corresponding authors.

## Abbreviations

FDR, false discovery rate; GSEA, gene set enrichment analysis; GWAS, genome-wide association studies; SNP, single-nucleotide polymophirsm; UV–DDB, ultraviolet radiation–DNA damage-binding protein; and XP, xedoderma pigmentosum.
