# Supplementary material for: Identification of New Biological Pathways Involved in Skin Aging From the Analysis of French Women Genome-Wide Data
Source: Front Genet. 2022 Mar 24;13:836581. doi: 10.3389/fgene.2022.836581 (PMC8987498; doi:10.3389/fgene.2022.836581)

Supplementary material

Identification of New Biological Pathways involved in Skin Aging from the analysis of French women genome-wide data

Supplementary table 1. Top ranked genes for the pathway reaching the 0.05 FDR threshold.

Phenotype	Pathway	Gene	SNP	SNP *P*-value
Wrinkling	Nucleotide excision repair	*XPC*	rs2733537	0.00153783
Wrinkling	Nucleotide excision repair	*MNAT1*	rs4151374	0.00519811
Wrinkling	Nucleotide excision repair	*DDB1*	rs2230356	0.00932987
Wrinkling	Proteasome	*PSMB3*	rs228275	0.000359898
Wrinkling	Proteasome	*PSMA1*	rs11023246	0.00118822
Wrinkling	Proteasome	*PSMC2*	rs10234363	0.00182129
Wrinkling	Proteasome	*PSMD2*	rs34881379	0.00257762
Wrinkling	Proteasome	*PSMB5*	rs12889177	0.00448658
Wrinkling	Proteasome	*PSMD4*	rs7489	0.00741838
Sagging	Nucleotide excision repair	*RAD23B*	rs16912372	0.000540118
Sagging	Nucleotide excision repair	*DDB1*	rs2230356	0.000670307
Sagging	Nucleotide excision repair	*ERCC8*	rs3117	0.00558406
Sagging	Nucleotide excision repair	*POLE4*	rs12713820	0.00670101
Sagging	mTOR signaling pathway	*RPS6KA2*	rs9347129	0.000175831
Sagging	mTOR signaling pathway	*PIK3R1*	rs251406	0.00194587
Sagging	mTOR signaling pathway	*TSC1*	rs7040593	0.00236903
Sagging	mTOR signaling pathway	*CAB39L*	rs2407616	0.00262206
Sagging	mTOR signaling pathway	*PIK3CA*	rs1607237	0.00282556
Sagging	mTOR signaling pathway	*RPTOR*	rs2048753	0.00316581
Sagging	mTOR signaling pathway	*VEGFA*	rs1570360	0.00717622
Sagging	Amino sugar and nucleotide sugar metabolism	*PMM2*	rs17747560	0.00167131
Sagging	Amino sugar and nucleotide sugar metabolism	*GFPT2*	rs6893651	0.00261209
Sagging	Amino sugar and nucleotide sugar metabolism	*GMDS*	rs3778565	0.00418288
Sagging	Amino sugar and nucleotide sugar metabolism	*CYB5R1*	rs4989513	0.00618583

Sagging	Amino sugar and nucleotide sugar metabolism	*CMAS*	rs728034	0.00702727

Supplementary table 2. Results of the pathway-based genome wide association study for photoaging.

Pathway name	Number of genes	*P*-value	FDR	Class	Subclass
Thyroid cancer	29	$1.4x10^{-3}$	0.11	Human Diseases	Cancers: Specific types
Primary immunodeficiency	31	$3.6x10^{-3}$	0.12	Human Diseases	Immune diseases
Selenoamino acid metabolism	26	$4.2x10^{-3}$	0.14	Metabolism	Metabolism of other amino acids
Wnt signaling pathway	145	$5x10^{-3}$	0.15	Environmental Information Processing	Signal transduction
Inositol phosphate metabolism	53	$5x10^{-3}$	0.16	Metabolism	Carbohydrate metabolism
Spliceosome	112	$8.6x10^{-3}$	0.17	Genetic Information Processing	Transcription
Nucleotide excsion and repair	38	$2.4x10^{-3}$	0.19	Genetic Information Processing	Replication and repair
Phosphatidylinositol signaling system	75	$5.2x10^{-3}$	0.19	Environmental Information Processing	Signal transduction
Long term potentiation	64	$8.4x10^{-3}$	0.19	Organismal Systems	Nervous system
Pyrimidine metabolism	85	$1.8x10^{-2}$	0.20	Metabolism	Nucleotide metabolism
Melanogenesis	100	$1.4x10^{-2}$	0.21	Organismal Systems	Endocrine system
Taste transduction	45	$1.6x10^{-2}$	0.22	Organismal Systems	Sensory system
Endocytosis	170	$1.3x10^{-2}$	0.22	Cellular Processes	Transport and catabolism
Proximal tubule bicarbonate reclamation	21	$2.5x10^{-2}$	0.24	Organismal Systems	Excretory system

FDR=False Discovery Rate,
The columns “Class” and “Subclass” describe the KEGG pathways classification

Supplementary table 3. Results of the pathway-based genome wide association study for solar lentigines.

Pathway name	Number of genes	*P*-value	FDR	Class	Subclass
Arginine and proline metabolism	45	$2.2x10^{-3}$	0.10	Metabolism	Amino acid metabolism
Thyroid cancer	29	$2x10^{-3}$	0.13	Human Diseases	Cancers: Specific types
Long term depression	67	$2x10^{-4}$	0.13	Organismal Systems	Nervous system
Cysteine and methionine metabolism	33	$2.4x10^{-3}$	0.13	Metabolism	Amino acid metabolism
Long term potentiation	64	$7.4x10^{-3}$	0.13	Organismal Systems	Nervous system
GnRH signaling pathway	96	$7.2x10^{-3}$	0.14	Organismal Systems	Endocrine system
Mismatch repair	21	$9.2x10^{-3}$	0.14	Genetic Information Processing	Replication and repair
Natural killer cell mediated cytotoxicity	118	$4.2x10^{-3}$	0.14	Organismal Systems	Immune system
Systemic lupus erythematosus	104	$1.1x10^{-2}$	0.15	Human Diseases	Immune diseases
Prion disease	32	$8.6x10^{-3}$	0.15	Human Diseases	Neurodegenerative diseases
Calcium signaling pathway	164	$6.4x10^{-3}$	0.15	Environmental Information Processing	Signal transduction
Gap junction	85	$5x10^{-3}$	0.15	Cellular Processes	Cellular community
Vasopressin regulated water reabsorption	41	$3.4x10^{-2}$	0.17	Organismal Systems	Excretory system
Glycine and serine and threonine metabolism	28	$2.9x10^{-2}$	0.20	Metabolism	Amino acid metabolism
B cell receptor signaling pathway	72	$2.5x10^{-2}$	0.20	Organismal Systems	Immune system
mTOR signaling pathway	49	$3x10^{-2}$	0.20	Environmental Information Processing	Signal transduction
Complement and coagulation cascades	66	$2x10^{-2}$	0.21	Organismal Systems	Immune system
Hypertrophic cardiomyopathy	80	$2.3x10^{-2}$	0.23	Human Diseases	Cardiovascular diseases
Phosphatidylinositol signaling system	75	$2.3x10^{-2}$	0.23	Environmental Information Processing	Signal transduction
Chemokine signaling pathway	174	$2.3x10^{-2}$	0.22	Organismal Systems	Immune system
Vascular smooth muscle contraction	109	$2.3x10^{-2}$	0.22	Organismal Systems	Circulatory system
Fc epsilon RI signaling pathway	77	$1.8x10^{-2}$	0.22	Organismal Systems	Immune system
Regulation of actin cytoskeleton	194	$2.2x10^{-2}$	0.23	Cellular Processes	Cell motility
melanogenesis	100	$2x10^{-2}$	0.24	Organismal Systems	Endocrine system
Arrhythmogenic right ventricular cardiomyopathy	71	$3.6x10^{-2}$	0.24	Human Diseases	Cardiovascular diseases

Type I diabetes mellitus	33	$4.3x10^{-2}$	0.24	Human Diseases	Endocrine and metabolic diseases

FDR=False Discovery Rate,
The columns “Class” and “Subclass” describe the KEGG pathways classification

Supplementary table 4. Results of the pathway-based genome wide association study for wrinkling.

Pathway name	Number of genes	*P*-value	FDR	Class	Subclass
Nucleotide excision and repair	38	$2x10^{-4}$	0.02	Genetic Information Processing	Replication and repair
Proteasome	42	$8x10^{-4}$	0.04	Genetic Information Processing	Folding, sorting and degradation
Bladder Cancer	39	$1x10^{-3}$	0.05	Human Diseases	Cancers: Specific types
Ribosome	76	$8.8x10^{-3}$	0.05	Genetic Information Processing	Translation
Primary immunodeficiency	31	$2.4x10^{-3}$	0.06	Human Diseases	Immune diseases
Purine metabolism	144	$6.4x10^{-3}$	0.18	Metabolism	Nucleotide metabolism
Vasopressin regulated water reabsorption	41	$8.4x10^{-3}$	0.18	Organismal Systems	Excretory system

FDR=False Discovery Rate,
The columns “Class” and “Subclass” describe the KEGG pathways classification

Supplementary table 5. Results of the pathway-based genome wide association study for sagging.

Pathway name	Number of genes	*P*-value	FDR	Class	Subclass
Amino sugar and nucleotide sugar metabolism	40	$2.2x10^{-4}$	0.05	Metabolism	Carbohydrate metabolism
mTOR signaling pathway	49	$8x10^{-4}$	0.05	Environmental Information Processing	Signal transduction
Nucleotide excision and repair	38	$2x10^{-4}$	0.05	Genetic Information Processing	Replication and repair
Ribosome	76	$1x10^{-2}$	0.13	Genetic Information Processing	Translation
FC gamma R mediated phagocytosis	90	$7.8x10^{-3}$	0.15	Organismal Systems	Immune system
O-glycan biosynthesis	29	$1.2x10^{-2}$	0.15	Metabolism	Glycan biosynthesis and metabolism
Cell cycle	118	$1x10^{-2}$	0.15	Cellular Processes	Cell growth and death
VEGF signaling pathway	75	$7.8x10^{-3}$	0.15	Environmental Information Processing	Signal transduction
DNA replication	33	$8x10^{-3}$	0.15	Genetic Information Processing	Replication and repair
Regulation of actin cytoskeleton	194	$9.8x10^{-3}$	0.15	Cellular Processes	Cell motility
Insulin signaling pathway	127	$6.8x10^{-3}$	0.16	Organismal Systems	Endocrine system
Small cell lung cancer	83	$8x10^{-3}$	0.16	Human Diseases	Cancers: Specific types
Adipocytokine signaling pathway	63	$1.4x10^{-2}$	0.16	Organismal Systems	Endocrine system
Basal transcription factor	29	$2.3x10^{-2}$	0.17	Genetic Information Processing	Transcription
Glycerolipid metabolism	43	$4.2x10^{-4}$	0.17	Metabolism	Lipid metabolism
PPAR signaling pathway	67	$2.2x10^{-2}$	0.17	Organismal Systems	Endocrine system
melanogenesis	100	$1.5x10^{-2}$	0.18	Organismal Systems	Endocrine system
Progesterone mediated oocyte maturation	78	$1.8x10^{-2}$	0.18	Organismal Systems	Endocrine system
Citrate cycle TCA cycle	28	$2.4x10^{-2}$	0.18	Metabolism	Carbohydrate metabolism
Arrhythmogenic right ventricular cardiomyopathy	71	$2.2x10^{-2}$	0.19	Human Diseases	Cardiovascular diseases
Primary immunodeficiency	31	$2.5x10^{-2}$	0.19	Human Diseases	Immune diseases
Dilated cardiomyopathy	85	$2.3x10^{-2}$	0.19	Human Diseases	Cardiovascular diseases
Instestinal immune network	39	$3.5x10^{-2}$	0.22	Organismal Systems	Immune diseases

FDR=False Discovery Rate,
The columns “Class” and “Subclass” describe the KEGG pathways classification

Supplementary table 6. Gene encoding different proteins which play a role in the same function in the "melanogenesis" pathway .

protein	genes	SNP	SNP *P*-value	pheno
Adenylate cyclase	*ADCY5*	rs4678003	$3.2x10^{-3}$	photoaging
	ADCY2	rs12155410	$5.6x10^{-3}$	lentigines
	ADCY1	rs16878777	$2.9x10^{-3}$	
	ADCY9	rs40996	$1.6x10^{-3}$	sagging
	ADCY2	rs13153439	$4.2x10^{-3}$	
	ADCY8	rs1028945	$9.7x10^{-3}$	
Protein Wnt	*WNT7B*	rs10448592	$7.6x10^{-4}$	photoaging
	WNT5B	rs1029628	$4.7x10^{-3}$	
	WNT7B	rs10453441	$7.1x10^{-4}$	lentigines
	WNT7B	rs10448600	$7.8x10^{-3}$	sagging
	WNT3A	rs3094913	$9.3x10^{-3}$	
Phospholipase C	*PLCB1*	rs6056223	$1.5x10^{-3}$	photoaging
	PLCB3	rs2244621	$1.8x10^{-3}$	lentigines
	PLCB4	rs2299683	$2.9x10^{-3}$	
	PLCB1	rs8114499	$3.6x10^{-3}$	sagging
	PLCB4	rs13044386	$4.1x10^{-3}$	
Calcium/calmodulin-dependent protein kinase	*CAMK2D*	rs13117519	$9.9x10^{-3}$	photoaging
	CAMK2G	rs11000785	$2.1x10^{-3}$	lentigines
	CAMK2D	rs13117519	$9.8x10^{-3}$	sagging
Protein kinase C	*PRKCB*	rs11865885	$4.0x10^{-4}$	photoaging
	PRKCA	rs17759657	$2.2x10^{-3}$ 0.00222545	
	PRKCA	rs9902960	0.00937863	lentigines
	PRKCB	rs11865885	0.00513123	sagging
	PRKCA	rs16959593	0.00976151	

Supplementary figure 1.

a. Melanogenesis pathway genes with SNPs associated with photoaging exhibiting *P*-values < 0.01 (green boxes). The star indicates several genes for the same protein function.

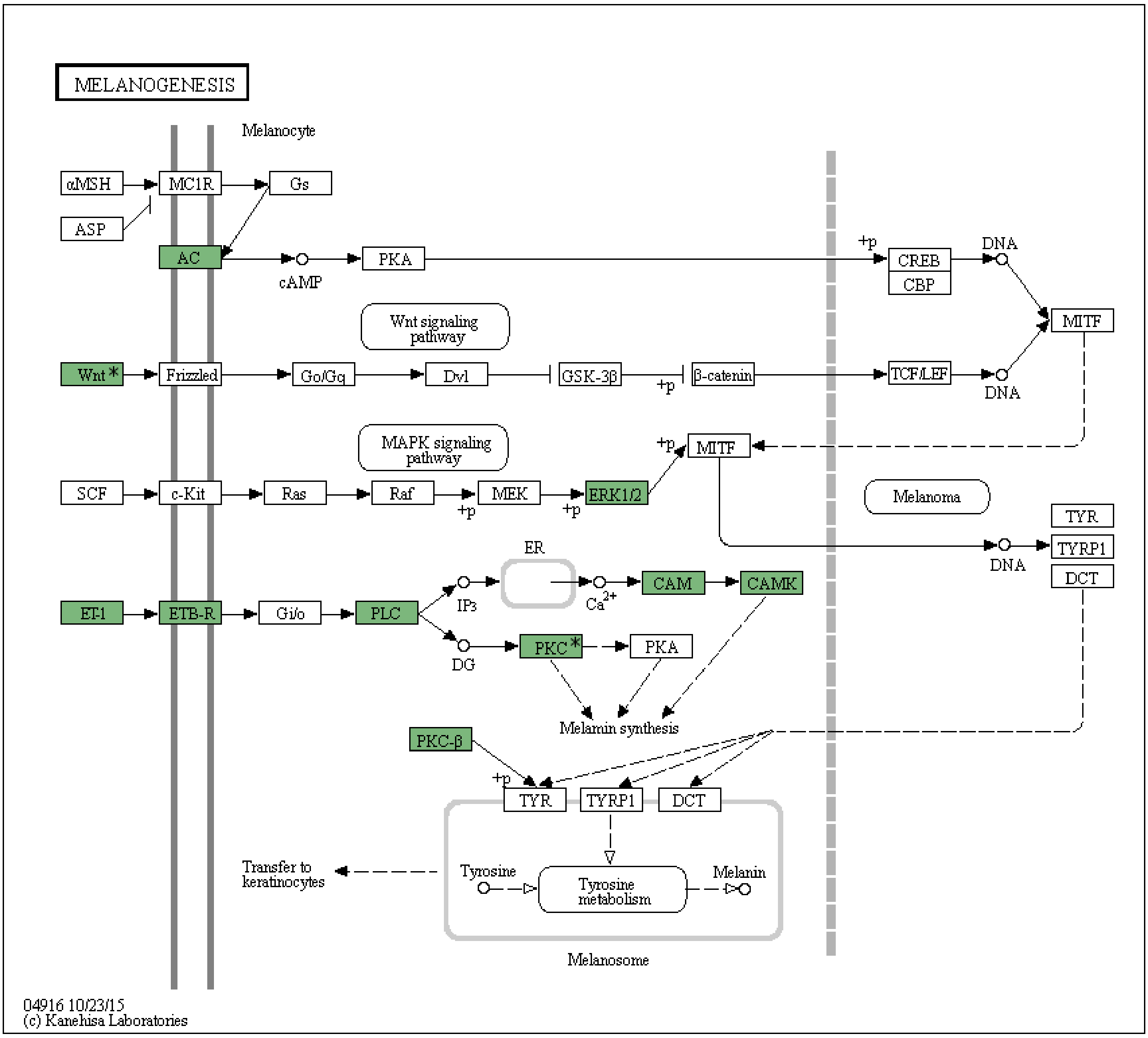

b. Melanogenesis pathway genes with SNPs associated with lentigines exhibiting *P*-values < 0.01 (green boxes). The star indicates several genes for the same protein function.

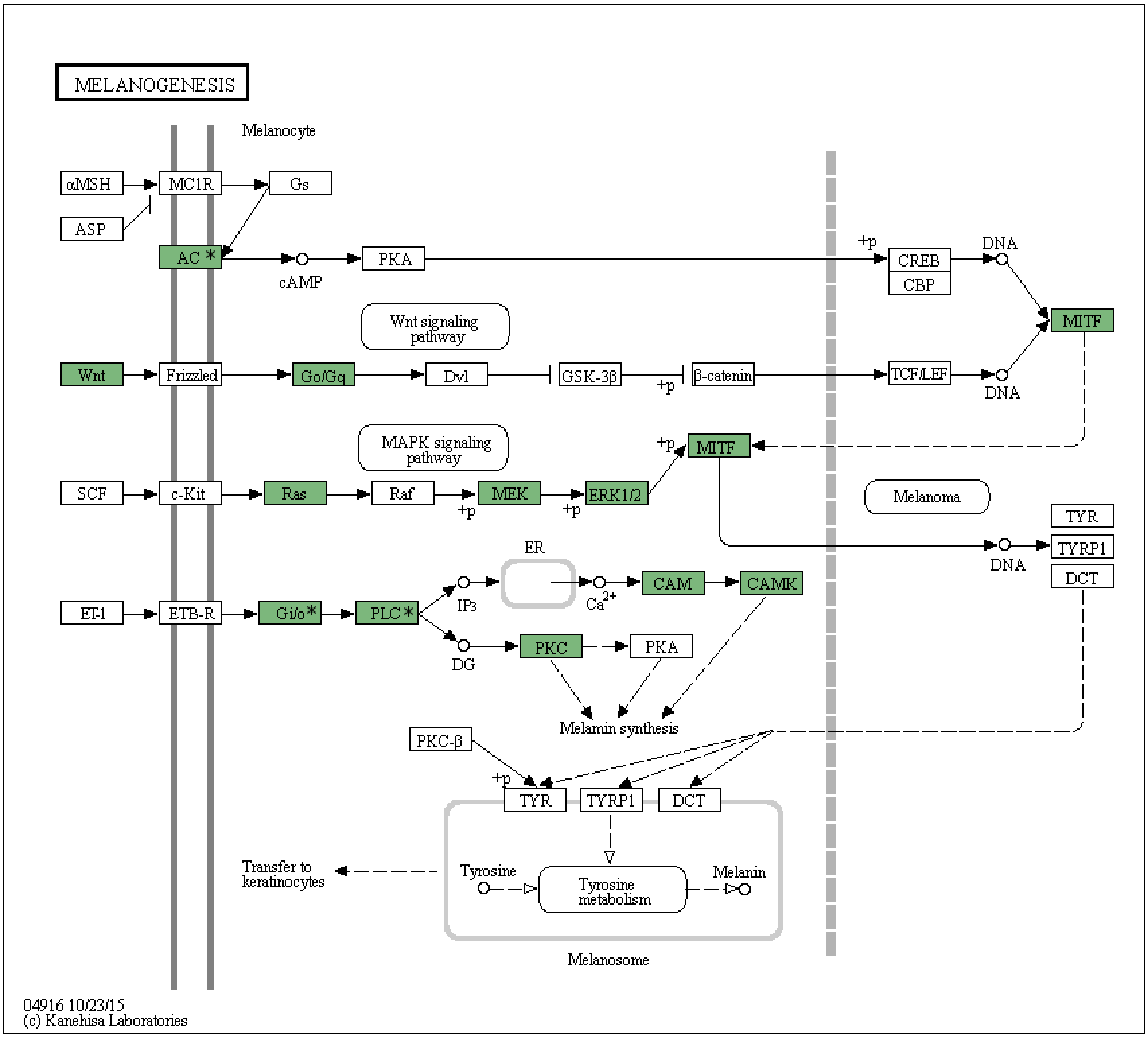

c. Melanogenesis pathway genes with SNPs associated with sagging exhibiting *P*-values < 0.01 (green boxes). The star indicates several genes for the same protein function.

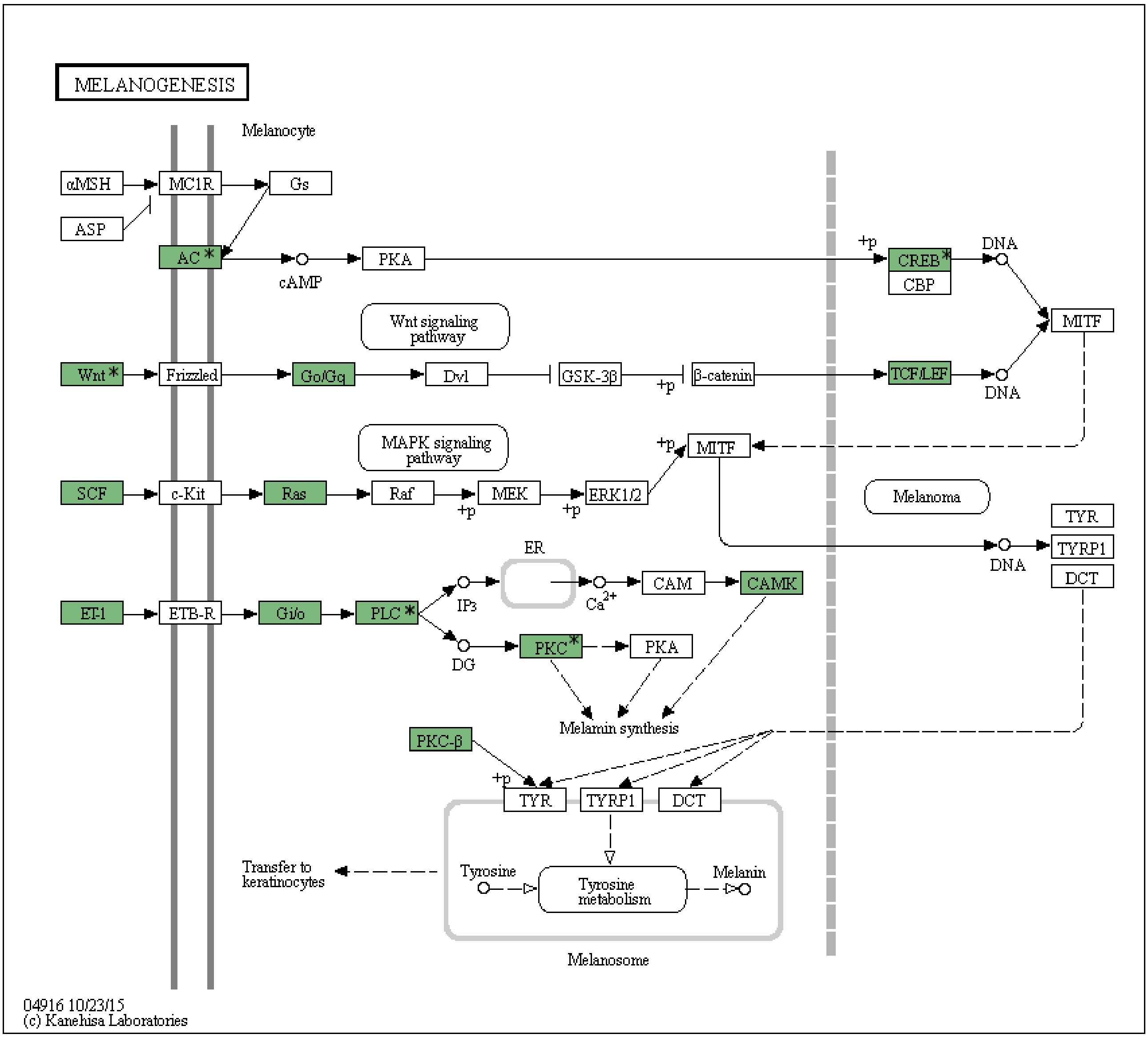

Supplementary figure 2.

a. Primary immunodeficiency pathway genes with SNPs associated with photoaging exhibiting *P*-values < 0.01 (green boxes).

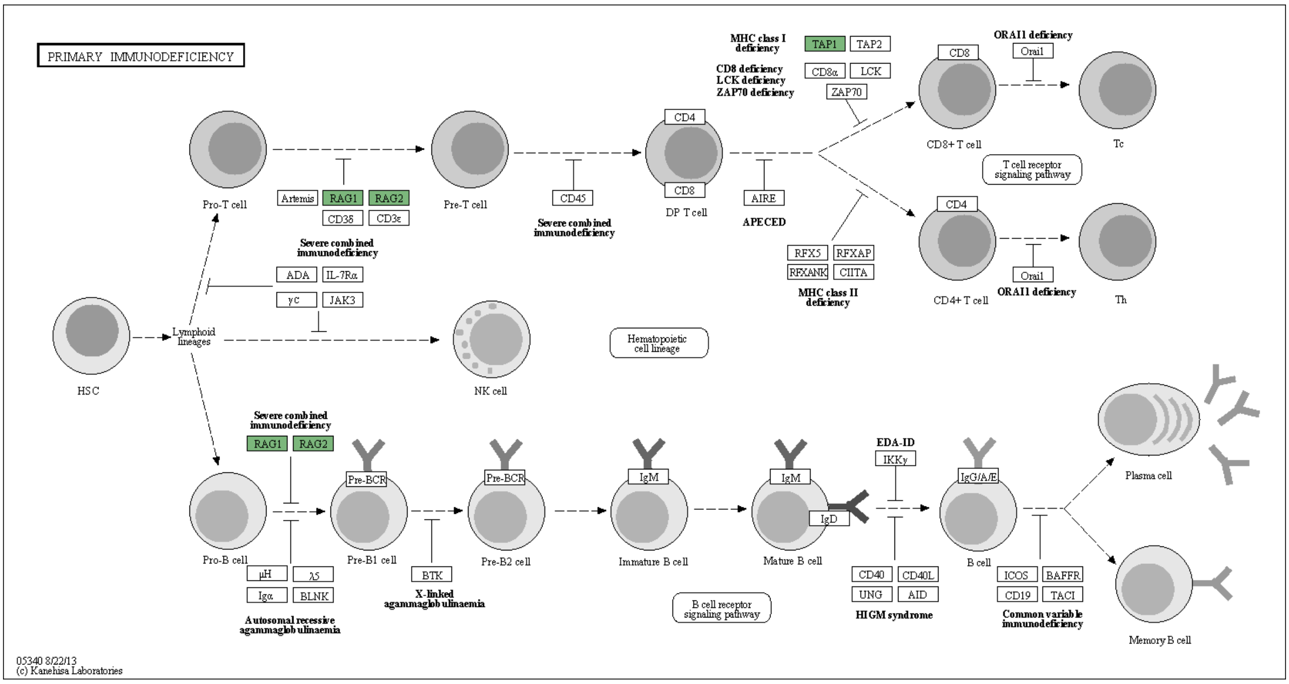

b. Primary immunodeficiency pathway genes with SNPs associated with sagging exhibiting *P*-values < 0.01 (green boxes).

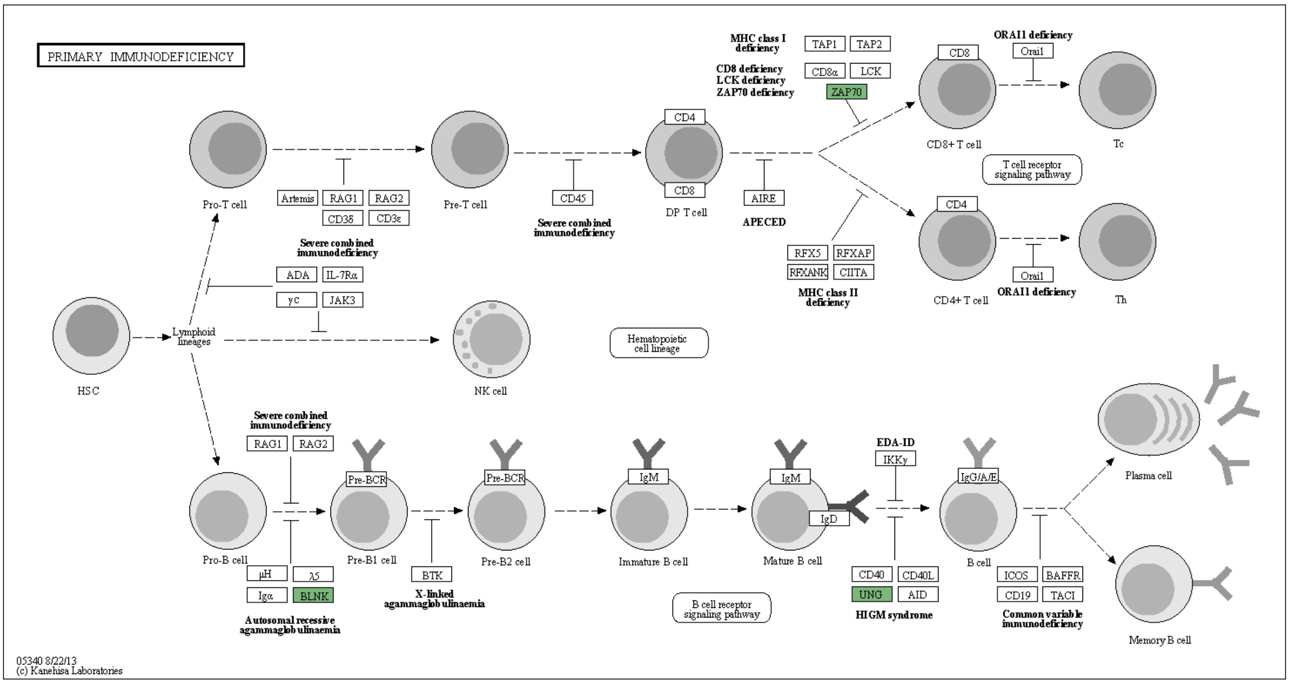

c. Primary immunodeficiency pathway genes with SNPs associated with wrinkling exhibiting *P*-values < 0.01 (green boxes).

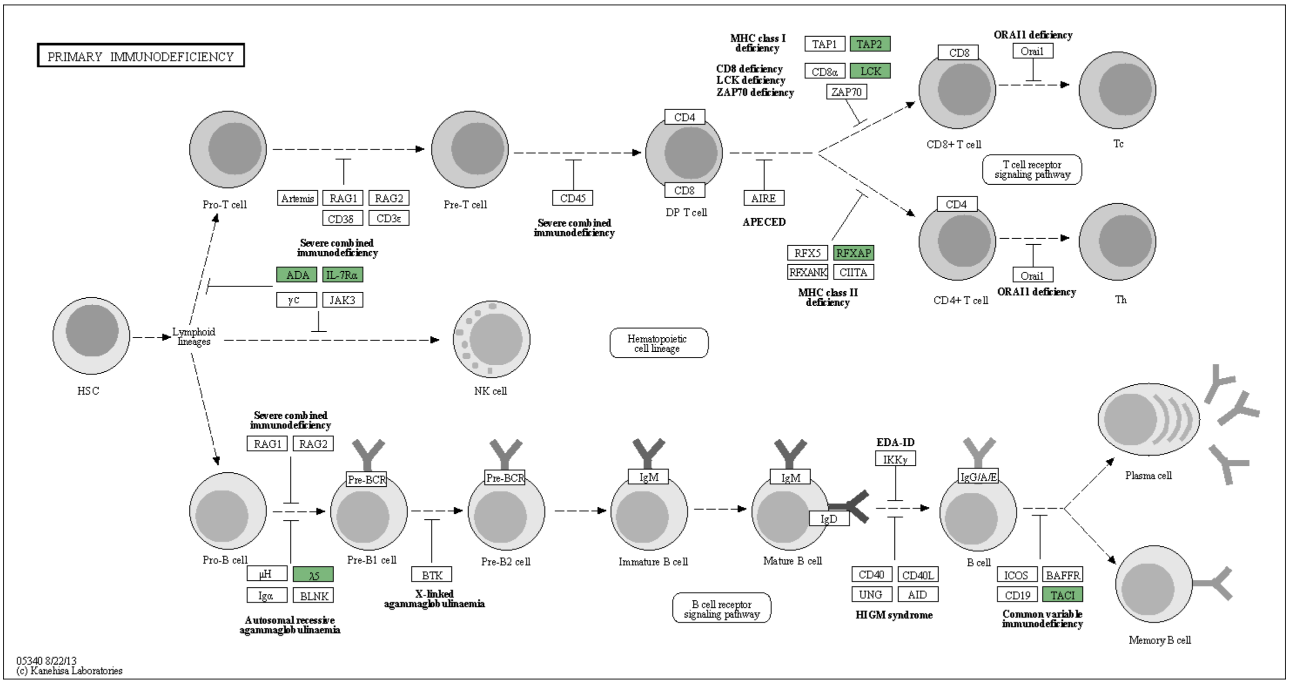

Supplementary figure 3. Distribution in percentage of pathways of interest (FDR < 0.25) for each outcome (photoaging, lentigines, sagging and wrinkling) in KEGG database categories. The categories distribution in KEGG was added as a reference

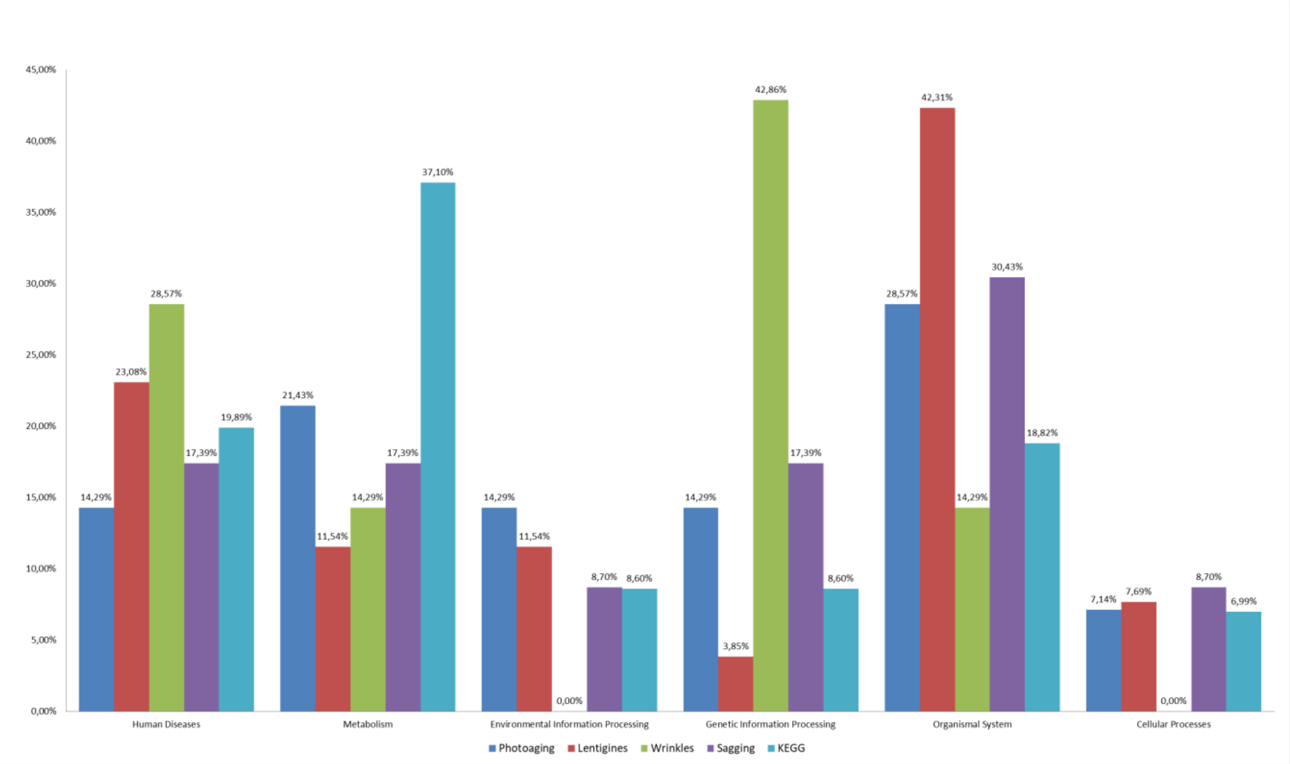

Supplement: Supplementary file 1 [file DataSheet1.PDF]
